# Monozygotic twins discordant for common variable immunodeficiency reveal impaired DNA demethylation during naïve-to-memory B-cell transition

**DOI:** 10.1038/ncomms8335

**Published:** 2015-06-17

**Authors:** Virginia C. Rodríguez-Cortez, Lucia del Pino-Molina, Javier Rodríguez-Ubreva, Laura Ciudad, David Gómez-Cabrero, Carlos Company, José M. Urquiza, Jesper Tegnér, Carlos Rodríguez-Gallego, Eduardo López-Granados, Esteban Ballestar

**Affiliations:** 1 Chromatin and Disease Group, Cancer Epigenetics and Biology Programme (PEBC), Bellvitge Biomedical Research Institute (IDIBELL), Avda Gran Via 199-203, 08908L'Hospitalet de Llobregat, Barcelona, Spain; 2 Department of Clinical Immunology, University Hospital La Paz, Paseo de la Castellana 261, 28046 Madrid, Spain; 3 Physiopathology of Lymphocytes in Immunodeficiencies Group, IdiPAZ Institute for Health Research, Paseo de la Castellana 261, 28046 Madrid, Spain; 4 Unit of Computational Medicine, Karolinska Institutet, Center for Molecular Medicine, Karolinska University Hospital, L8:05, SE-171 76 Stockholm, Sweden; 5 Department of Immunology, University Hospital Son Espases, Carretera de Valldemossa, 79, 07120 Palma de Mallorca, Spain

## Abstract

Common variable immunodeficiency (CVID), the most frequent primary immunodeficiency characterized by loss of B-cell function, depends partly on genetic defects, and epigenetic changes are thought to contribute to its aetiology. Here we perform a high-throughput DNA methylation analysis of this disorder using a pair of CVID-discordant MZ twins and show predominant gain of DNA methylation in CVID B cells with respect to those from the healthy sibling in critical B lymphocyte genes, such as *PIK3CD*, *BCL2L1*, *RPS6KB2*, *TCF3* and *KCNN4*. Individual analysis confirms hypermethylation of these genes. Analysis in naive, unswitched and switched memory B cells in a CVID patient cohort shows impaired ability to demethylate and upregulate these genes in transitioning from naive to memory cells in CVID. Our results not only indicate a role for epigenetic alterations in CVID but also identify relevant DNA methylation changes in B cells that could explain the clinical manifestations of CVID individuals.

## Abstract (web summary)

Epigenetic changes are thought to contribute to the aetiology of common variable immunodeficiency (CVID), a disease characterized by loss of B-cell function. Here, by comparing DNA methylation profile in B cells from monozygotic twins discordant for CVID, the authors show a gain of DNA methylation in CVID B cells.

## Graphical abstract

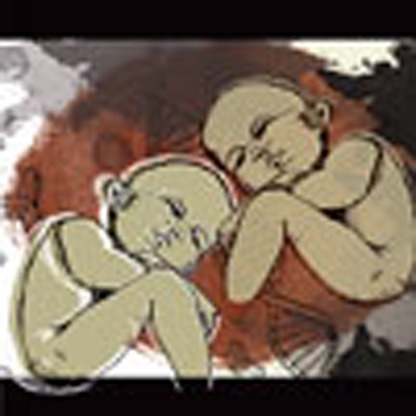

Common variable immunodeficiency (CVID) is the most frequent symptomatic primary immunodeficiency (PID) in adults. It comprises a heterogeneous group of disorders that are characterized by hypogammaglobulinaemia that predisposes CVID individuals to recurrent bacterial infections that mainly affect the respiratory and gastrointestinal tracts^1^. CVID patients present a marked decrease in IgG and at least one of the isotypes IgM or IgA. Other criteria used for diagnosis include the onset of immunodeficiency after the age of 2 years, a poor specific antibody response and exclusion of known monogenic causes of hypogammaglobulinaemia^2^. The clinical manifestations of CVID are highly variable. Several complications, such as lymphoproliferative disorders and inflammatory diseases, occur in some CVID patients. In addition, over 25% of CVID cases present autoimmune manifestations. These complications are poorly understood and sometimes associated with poor prognosis^3^.

CVID is usually detected sporadically in patients with no family history of immunodeficiency. However, for ∼10% of subjects other first-degree relatives may be either hypogammaglobulinaemic or present selective IgA deficiency^4^,^5^. In fact, progression from selective IgA deficiency to CVID has been observed in several patients. Although CVID is considered to be an intrinsic defect, it can be clinically developed at any age suggesting cumulative deterioration of B-cell functions in a putative multifactorial pathogenic process. In this sense, comparing genetically identical monozygotic (MZ) twins concordant or discordant for CVID could be a useful strategy for dissecting the genetic and non-genetic components of this disease. Enormous efforts are made to find genetic defects that would help understand CVID aetiology and predict the clinical outcome. However, genetic alterations account for the condition in only ∼10–20% of immunodeficiency patients, and the majority lack a definite molecular genetic diagnosis^6^. Genetic mutations can affect several immune processes, especially those related to B-cell activation, T-cell signalling and cytokine expression^6^. For instance, there are mutations in the genes encoding CD19, CD21, CD81, PLC-γ2, which are proteins involved in signalling through the B-cell receptor (BCR), and in those encoding ICOS, BAFFR, TACI, MSH5, involved in co-stimulatory pathways necessary for isotype switching and somatic hypermutation during B-cell activation^7^. Analysis of consanguineous families affected by hypogammaglobulinaemia has led to identifying new mutations in genes such as *LRBA*^8^. In addition, mutations that cause loss and gain of function have been identified in genes such as *CTLA4* (ref. 9) and *PIK3CD*^10^, respectively.

CVID is characterized by deregulation of various immune cell types. Examples include reduction of CD4 T cells and increase in CD8 T cells^11^, and reductions in both myeloid and plasmacytoid dendritic cell frequencies^12^. However, the most uniform alteration is observed in the B-cell compartment. Specifically, CVID patients display a severe deficiency of switched memory B cells CD27+IgD−IgM− (ref. 13) and, in fact, it has been proposed to subclassify patients based on the distribution of naive/memory B cells defined by the expression of IgD and CD27 (ref. 14). Moreover, the expansion of certain B-cell subsets, such as CD21^lo^ or transitional B cells, correlates with complications such as splenomegaly and lymphadenopathy, respectively, while the reduction of class-switched memory B cells has been linked with granulomatous disease and autoimmune cytopenias^15^.

During B-cell differentiation, identity is acquired through significant changes in gene expression. These changes are tightly regulated, not only by successive networks of transcription factors but also by epigenetic mechanisms, including DNA methylation^16^,^17^. DNA methylation plays a fundamental role in differentiation by driving and stabilizing gene activity states during cell fate decisions. Methylation takes place in cytosines located in CpG dinucleotides. CpGs in different regions play a regulatory role, including those in CpG islands, which are CpG-dense regions, ‘shores', located 1–2 kb upstream or downstream of CpG islands, and ‘shelves', adjacent to shores^18^,^19^. Promoters and enhancers are common hotspots for functionally relevant DNA methylation changes. Thus, DNA methylation is important in processes such as the maintenance of chromosome stability, genomic imprinting and regulation of gene expression^20^. In fact, a DNA demethylation signature has been described in both early stages of B-cell development^21^ and late stages, during activation of naive B cells^22^. In addition, profiling of the DNA methylome and transcriptome of B lymphocyte subsets representing stages of the humoral response before and after antigen exposure *in vivo* reveals the generation of DNA methylation profiles that map to transcription-binding sites and that are retained in the progeny of activated B cells, generating a similar epigenetic signature in downstream memory cells and plasma cells, with distinct transcriptional programmes^22^.

Direct comparison of the DNA methylation patterns in cells from identical twins is an excellent experimental approach for testing the contribution of epigenetic changes to complex diseases because DNA sequence differences, including single-nucleotide polymorphisms, do not interfere with such an analysis. Recent studies with twins in the context of immune-related disease^23^,^24^,^25^ have demonstrated the existence of genome-wide epigenetic differences that could explain differences in phenotype. Given the importance of B cells in CVID aetiology and the influence of DNA methylation on the normal function and development of this cell type, in the work reported here, we initially compared the DNA methylation profiles of B cells of a single MZ twin pair discordant for CVID. The comparison revealed significant changes in DNA methylation associated with CVID, specifically the hypermethylation of several genes of relevance in B-cell biology, including *PIK3CD*, *BCL2L1*, *RPS6KB2*, *TCF3* and *KCNN4*. The comparison of the DNA methylation status of selected genes from the above screening in naive, unswitched and switched memory B cells from a cohort of healthy versus CVID individuals revealed that the aforementioned hypermethylation does not affect the naive cell subpopulation. However, we observed that CVID individuals were impaired for the demethylation and upregulation of these genes in the transition from naive to unswitched and switched memory cells. Our results reveal a novel mechanism by which the acquisition of the epigenetic signature in memory cells in CVID individuals is impaired, perhaps accounting for their decreased survival in CVID patients, and suggesting potential targets for clinical intervention.

## Results

### CVID-discordant twins reveal DNA methylation changes

**Figure 1:**
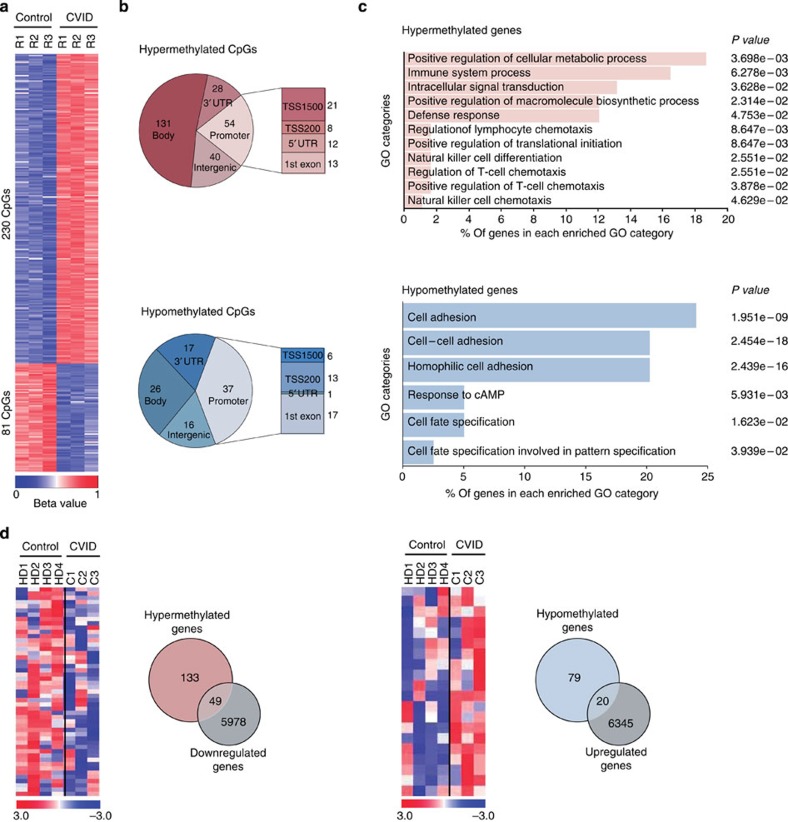
Comparison of B-cell DNA methylation profiles from MZ twins discordant for CVID. (**a**) Heatmap showing differentially methylated CpGs between CD19+B cells from twins discordant for CVID obtained from the DNA methylation array. A technical triplicate was analysed for each sample (R1, R2 and R3). The heatmap scale shows the range of *β*-values, whereby positive (red) and negative (blue) values correspond, respectively, to a higher and a lower methylation status than average. B cells from CVID twin showed 230 hypermethylated CpGs, while 81 CpGs were hypomethylated (Δ*β*-value≥0.1). (**b**) Genomic distribution of differentially methylated CpGs. The analysed CpGs could be associated with genes and localized in the 3′ UTR, gene body or in the promoter region. At the promoter level, the CpGs could be localized within 1,500 bp of the transcription start site (TSS1500), within 200 bp of the transcription start site (TSS200), in the 5′ UTR or in the first exon. The CpGs that are not associated with genes are considered intergenic. (**c**) Gene ontology enrichment analysis of genes associated with differentially methylated CpGs. The bar charts show the most relevant and significantly enriched GO categories, the *P* values and the percentage of hypermethylated or hypomethylated genes in each category. (**d**) Heatmaps showing the expression differences between a small cohort of CVID and healthy individuals for hypermethylated and hypomethylated genes (left and right panels, respectively). The heatmap scale shows the range of expression values, whereby positive (red) and negative (blue) values correspond, respectively, to a higher and a lower expression status than average. On the right of each heatmap, a Venn diagram shows the overlap between genes that are hypermethylated and genes that are less strongly expressed in CVID with respect to healthy individuals or hypomethylated and genes that are expressed at higher levels in CVID with respect to healthy individuals.

To investigate the potential occurrence of DNA methylation alterations in B cells of CVID individuals, we first performed DNA methylation screening on CD19+ cells isolated from a pair of MZ twins discordant for CVID. One of the individuals had been diagnosed with archetypical CVID, with a virtual absence of switched memory B cells, whereas his healthy sibling only presented moderately reduced levels of IgA, with IgG that had been increasing to normal levels with age. For the analysis, we used bead arrays to interrogate the DNA methylation status of >450,000 CpG sites across the entire genome covering 99% of RefSeq genes. Samples were processed and analysed in triplicate. We observed that array-wide technical variability (three replicates per twin: variability within a twin) and biological variability (differences between twins) were similar. For this reason we designed a robust CpG criterion selection (see Bioinformatics analysis in Methods) in order to identify differentially methylated candidate regions that were profiled later replication in an independent replication cohort. By the selection procedure we identified 311 CpG sites that were differentially DNA-methylated between the CVID individual and his healthy matching twin (Supplementary Data 1). Given the small range of changes, we considered a CpG to be differentially methylated when the difference in β was greater than 10%. Approximately 75% of these differentially methylated CpGs (230 out of 311) displayed higher methylation levels in the CVID individual than in his sibling, and the remaining 25% (81 CpGs) were less methylated in the CVID compared with the healthy sibling (Fig. 1a). The majority of these changes occurred in annotated genes (79.1% and 80.2% in the hypermethylated and hypomethylated sets, respectively; Fig. 1b). Of the gene-associated hypermethylated CpGs, 131 were located in gene bodies, 28 at the 3′ untranslated region (UTR) and 54 at gene promoters. In the hypomethylated set of CpGs, 26 CpGs were located in gene bodies, 17 CpGs at 3′ UTR and 37 in promoter regions (Fig. 1b).

We then performed gene ontology (GO) analysis to determine whether the differentially methylated genes were associated with potentially relevant biological processes in CVID. We independently analysed the lists of hypermethylated and hypomethylated genes. In the hypermethylated set of genes, there was enrichment of important GO categories such as immune system process (GO:0002376), intracellular signal transduction (GO:0035556), defense response (GO:0006952), positive regulation of macromolecule biosynthetic process (GO:0010557) and positive regulation of cellular metabolic process (GO:0031325). We found 12–19% of the hypermethylated genes to be associated with these categories. A smaller percentage of hypermethylated genes was associated with other interesting categories in CVID such as regulation of lymphocyte chemotaxis (GO:1901623), or positive regulation of T-cell chemotaxis (GO:0010820). In the hypomethylated group, the functional categories were less obviously relevant in the context of B-cell biology. We found that 24% of genes were associated with cell adhesion (GO:0007155), 20% were in the cell–cell adhesion (GO:0098609) and homophilic cell adhesion (GO:0007156) categories. Around 3–5% of hypomethylated genes were associated with other categories, such as response to cAMP (GO:0051591), cell fate specification involved in pattern specification (GO:0060573) and cell fate specification (GO:0001708; Fig. 1c).

The hypermethylated genes (Table 1) were particularly interesting for their relevance in BCR signalling pathways such as: PI3K signalling in B lymphocytes, FcRIIB signalling in B lymphocytes, CD27 signalling, P38MAPK, CD40 signalling, NF-KB signalling, APRIL-mediated signalling, B-cell-activating factor signalling, pathway of inositol phosphate compounds. All these genes have associated network functions that the programme identified as: cell death and survival, cell-mediated immune response and cellular movement.

In addition to the overall analysis of functional categories, inspection of individual genes made it possible to identify those essential for B-cell biology and function from among the list of hypermethylated genes. These included genes such as *PIK3CD*, *TCF3*, *KCNN4, BCL2L1* and *RPS6KB2*, among others (Table 1). *PIK3CD* encodes the catalytic subunit delta of the phosphatidylinositol-4,5-bisphosphate 3-kinase, a molecule that regulates natural antibody production, marginal zone and B-1 B-cell function, and autoantibody responses^26^. Another interesting example is *KCNN4*, a molecule that is activated during B-cell activation and helps maintain elevated Ca^2+^ levels during signal transduction^27^. *BCL2L1* encodes an apoptotic inhibitor that plays a critical role in the germinal centre^28^. The list of hypomethylated genes had less obvious candidates in terms of B-cell biology.

We then compared the DNA methylation data with existing gene expression data obtained from B cells of CVID patients versus healthy donors (accession no. GSE17269)^29^ (Fig. 1d). Despite the differences between this study focusing on a small cohort and also using the naive CD19+ subset, we found a significant number of genes with differences in expression among those displaying differences in DNA methylation (Fig. 1d). This suggests that the methylation changes occurring in CVID B cells may affect the overall expression of the associated genes.

### DNA methylation changes in CVID are B-cell-specific

**Figure 2:**
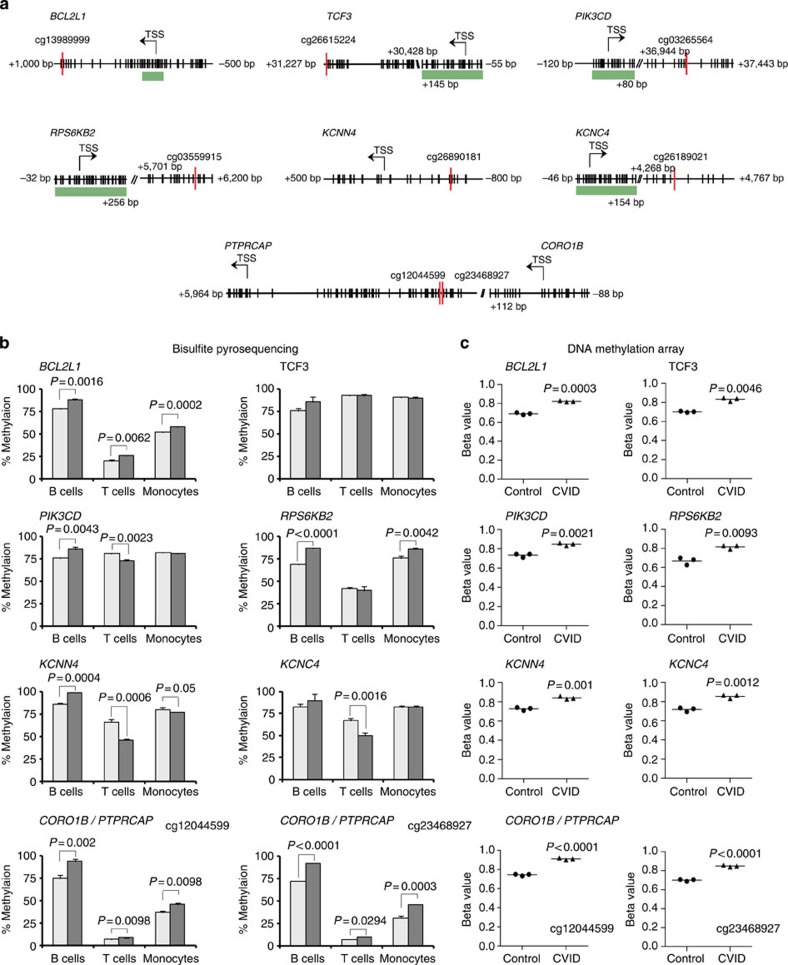
Validation of DNA methylation results by bisulfite pyrosequencing. (**a**) Schemes depicting a selection of genes indicating the differentially methylated CpG site (red line) and its relative location with respect to the TSS (located with an arrow). Additional CpG sites are represented with vertical black lines. CpG islands are represented with a green bar. (**b**) Bisulfite pyrosequencing of selected genes, all of which are relevant to B-cell biology. In addition to CD19+ B lymphocytes, CD4+ T lymphocytes and CD14+ cells (monocytes) were analysed for all genes. Methylation levels are represented as percentages. Bisulfite sequencing was technical triplicates of each sibling of the MWZ twin pair. Error bars correspond to s.d. Student's *t*-test comparisons with a *P* value above 0.05 are considered with a nonsignificant difference, and are not presented (**c**) *β*-values obtained from the DNA methylation array. The B cells from CVID twin have higher levels of DNA methylation (Δ*β*≥0.1) in the analysed genes than those from his healthy sibling. Black filled circles and black filled triangles, respectively, indicate the three *β*-values for the control twin and the CVID twin. The black line indicates the average of the *β*-values in each condition.

To confirm the results obtained from the DNA methylation arrays, we performed bisulfite pyrosequencing of a selection of the aforementioned genes (Table 1), hypermethylated in B cells isolated from the same pair of twins discordant for CVID. In brief, the selection of genes was carried out among those displaying the largest changes in DNA methylation between the CVID and healthy sibling, and also to cover different functions in B-cell biology: *PIK3CD*, *RPS6KB2* encode proteins involved in the cell proliferation, cell growth and protein synthesis, that occur in response to BCR signalling; *BCL2L1* encodes a protein with anti-apoptotic effects. *TCF3* encodes a transcription factor essential in the establishment of B-cell identity. *PTPRCAP* encodes a protein necessary for proper phosphatase activity of CD45 during BCR signalling, and *CORO1B*, *KCNN4* and *KCNC4* encode proteins that are involved in Ca+2 signalling. We focused on looking at the same CpG sites for which significant changes had been identified (Fig. 2a). Despite the modest differences in DNA methylation measured by the initial high-throughput strategy, we confirmed by bisulfite pyrosequencing a robust increase in DNA methylation in the CVID sibling with respect to the healthy sibling (Fig. 2b), with very similar values to those obtained using bead arrays (Fig. 2c). We also tested the DNA methylation changes in CD4+ cells (T lymphocytes) and CD14+ cells (monocytes) from the same individuals (Fig. 2b). In contrast with the robust increase in the frequency of B cells from the CVID sibling with respect to the healthy sibling, we observed no differences or changes in the opposite direction in T cells and monocytes, highlighting the specificity of the observed CVID-associated hypermethylation of these genes to B cells.

As mentioned earlier, CVID individuals have been reported to contain fewer switched memory cells. Our results showing DNA methylation differences between this CVID individual and his matching healthy sibling using total CD19+ may simply reflect changes in the proportion of different B-cell subsets between these two individuals if the DNA methylation levels for these genes were different for naive, unswitched and switched memory B cells. For this reason, it is essential to analyse DNA methylation using separate B-cell subsets, to distinguish between the change in B-cell subset proportions and the existence of *bona fide* changes in the DNA methylation status of these genes in specific B-cell subpopulations.

### Methylation impaired in naive-to-memory B cell transition

**Figure 3:**
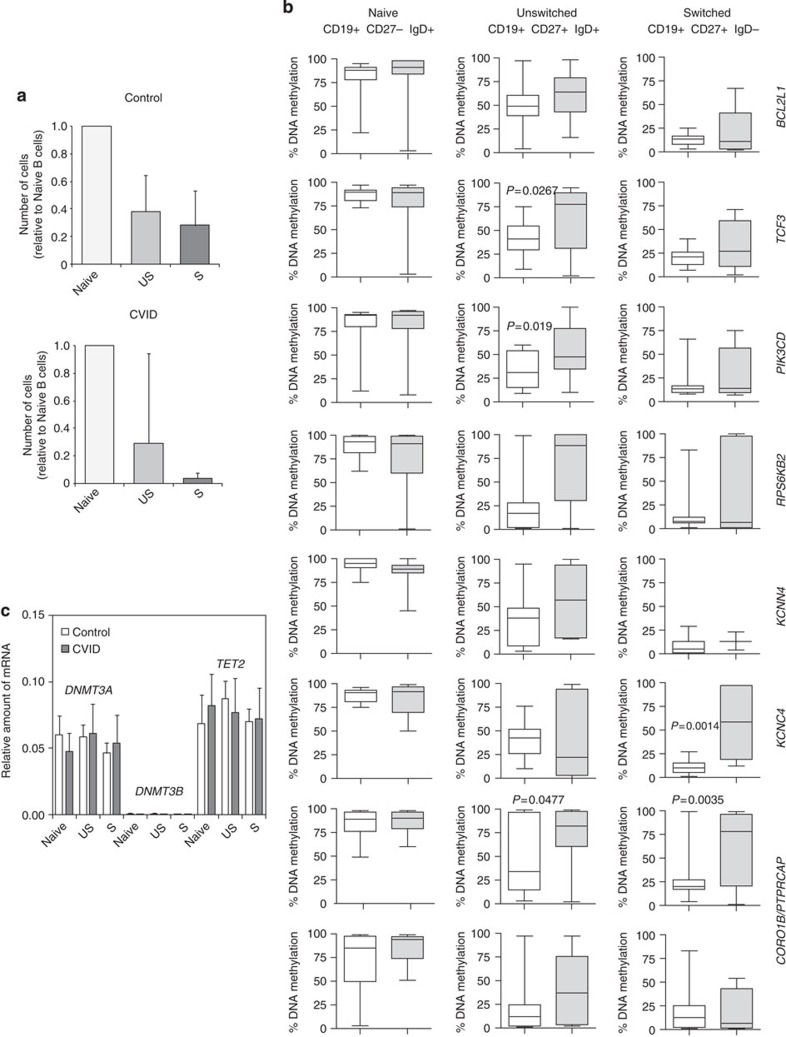
Comparison of DNA methylation levels of selected genes in different B-cell subsets between CVID patients and healthy donors. (**a**) Graph showing the relative number of cells in each B-cell subset (naive, unswitched (US) and switched (S) memory cells) in CVID patients and healthy donors. The cell number is relative to the naive B-cell subset. Error bars correspond to s.e.m. (**b**) Box and whisker plots showing the percentage of DNA methylation obtained by pyrosequencing in the eight validated genes. White boxes represent the DNA methylation levels in healthy donors. Grey boxes represent DNA methylation levels in CVID patients. The analysed B-cell subsets were naive, unswitched and switched B cells. The *P* value is shown for the cases with a statistically significant difference. The bottom and top of the box are the first and third quartiles, respectively, the band inside the box is the median and the ends of the whiskers represent the minimum and maximum of all of the data. Student's *t*-test comparisons with a *P* value above 0.05 are considered with a nonsignificant difference, and are not presented. (**c**) Quantitative RT–PCR analysis comparing mRNA levels of *DNMT3A*, *DNMT3B* and *TET2* in different B-cell subsets in five CVID patients and four healthy controls. *HPRT1* was used for normalization. In this analysis, we compared five CVID patients and five controls. Error bars correspond to s.e.m.

To address the aforementioned question, we therefore proceeded to analyse the DNA methylation levels in selected hypermethylated genes for three B-cell subsets in a cohort of 12 healthy donors and 16 CVID patients. Specifically, we measured the methylation levels of all these genes in naive (CD19+CD27−IgD+), unswitched (CD19+CD27+IgD+) and switched (CD19+CD27+IgD−) memory B lymphocytes. The relative numbers of these B-cell subsets differed between CVID and healthy individuals. For an equivalent number of naive cells, the unswitched B-cell subset was present at similar levels in CVID and healthy individuals; however, switched memory cells were much less frequent in CVID individuals (Fig. 3a).

We then compared the methylation levels of the B-cell subsets in the control group, observing a progressive loss of methylation from naive cells to switched memory B cells (Fig. 3b and Supplementary Data 2). For instance, *BCL2L1* displayed an average 79% methylation in naive cells, 49% in unswitched memory cells and only 13% in switched memory cells (Table 2). Similar changes towards lower DNA methylation levels from naive-to-switched memory cells were found for all the other genes in our list (Table 2), highlighting the close association between the differentiation of naive cells to memory cells and the loss of methylation in these relevant genes. DNA demethylation appears to be independent of the global levels of enzymes involved in their deposition (such as DNMT3a and DNMT3B) or removal (such as TET2), as suggested by the absence of changes during naive-to-memory B-cell differentiation (Fig. 3c).

**Figure 4:**
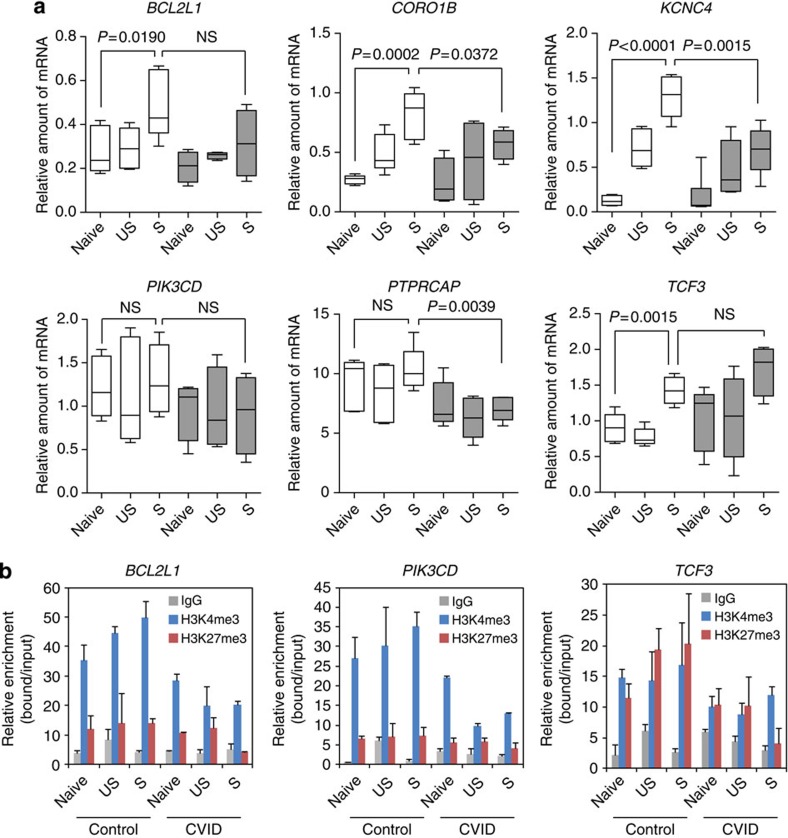
Comparison of the expression and histone modification levels of aforementioned selected genes in different B-cell subsets in healthy donors and CVID patients. (**a**) Box and whisker plots showing quantitative RT–PCR analysis comparing mRNA levels of the selected genes in different B-cell subsets in five CVID patients and four healthy controls. *HPRT1* was used for normalization. Grey boxes represent expression levels in CVID patients. The bottom and top of the box are the first and third quartiles, respectively, the band inside the box is the median and the ends of the whiskers represent the minimum and maximum of all of the data. Student's *t*-test comparisons with a *P* value above 0.05 are considered with a nonsignificant difference (n.s.). (**b**) ChIP assays for two histone modifications, one activating marks (H3K4me3, blue bars) and one repressive mark (H3K27me3, red bars) focusing on the same regions were changes in DNA methylation were identified. The values correspond to relative enrichment of the bound fraction with respect to input. Negative control (immunoprecipitated with IgG) samples are also shown. The comparison corresponds to a technical triplicate of one CVID individual and one healthy control. Error bars correspond to s.d.

Demethylation of these genes is compatible with the increased expression levels of these genes and the function of their products in memory cells. These results are also in agreement with the progressive loss of methylation and gain of expression during B-cell differentiation^22^. We used quantitative real-time PCR (QRT–PCR) to measure mRNA levels of these genes, and observed that normal individuals exhibit increasing levels of genes such as *BCL2L1*, *KCNC4* and *CORO1B* in the naive-to-switched memory B-cell transition, confirming a relationship between DNA demethylation and gain of expression (Fig. 4a). In fact, analysis of histone modifications in the sequences containing the CpG sites undergoing demethylation showed an increase in the activating histone mark H3K4me3 during naive to memory cell differentiation in genes such as *BCL2L1* and *PIK3CD* (Fig. 4b), indicating a relationship between DNA methylation, histone modifications and gene expression and a less clear correlation for the repressive mark H3K27me3 (Fig. 4b).

Most importantly, when we analysed the DNA methylation levels of these genes in the three B-cell subsets in CVID patients, we observed impairment in the loss of DNA methylation as cells transition from naive to switched memory B cells (Fig. 3b). For instance, focusing again on *BCL2L1*, the average methylation levels were significantly higher for memory cells (both unswitched and switched) in CVID individuals than in healthy controls (Table 2). This behaviour was generally observed for all genes (see Fig. 3b and Table 2). Analysis of variance (ANOVA) test confirmed the generality of these findings across the panel of genes analysed (see Supplementary Table 1). We observed that the levels of methylation for all genes in naive B cells from CVID individuals were almost identical to those from healthy controls. However, the average levels for all these genes in unswitched and switched B cells were higher for CVID than for control individuals. In general, unswitched and switched memory B cells tended to be hypermethylated relative to controls. This finding suggests impaired loss of methylation for these genes during B-cell differentiation in CVID patients. These differences in DNA demethylation between CVID individuals and healthy controls are not related to changes in the global levels in DNMTs or TET proteins (Fig. 3c).

Regarding mRNA levels, we observed that CVID individuals display altered levels in both unswitched and switched memory cells, confirming the relevance of the observed impaired DNA demethylation during differentiation to memory B cells (Fig. 4a). For instance, *BCL2L1*, *KCNC4* and *CORO1B* display lower levels of expression in CVID individuals, supporting a functional consequence of their impairment in DNA demethylation. In addition, analysis of histone modifications H3K4me3 for some of these sequences revealed lower levels in CVID individuals when compared with healthy controls for genes such as *BCL2L1* or *PIK3CD* (Fig. 4b).

## Discussion

Our study demonstrates for the first time the existence of DNA methylation alterations in CVID, most notably, in genes relevant to B-cell function. The observed changes in CVID individuals indicate that the unswitched and switched memory cell subsets are impaired to achieve the degree of DNA demethylation found in healthy individuals. In contrast, the naive B-cell subset of CVID patients displays normal levels of methylation. The observed changes suggest a potential mechanism participating in the defective generation of memory cells in CVID individuals. In addition, our results prove that studies of MZ twins are extremely valuable for identifying DNA methylation differences, since interference with the genetic contribution is minimized and it is possible to tease out the epigenetic component.

**Figure 5:**
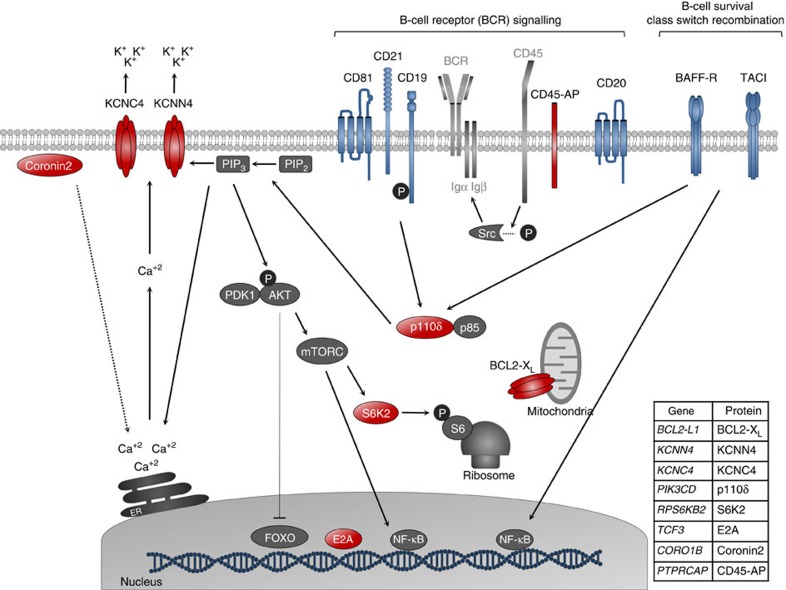
Scheme depicting B-cell factors implicated in CVID, from genetic studies, or displaying DNA methylation alterations (from this study). Molecules whose encoding genes have been reported to be altered in CVID are shown in blue. The products of genes that were found to be hypermethylated in memory B cells in our study are shown in red. Molecules important in B-cell biology and related to the genetically or epigenetically altered molecules are shown in grey. The table in the right bottom panel contains the names of the genes and proteins. The following proteins/genes are also represented: BCR, B-cell receptor; Igα and Igβ, BCR co-receptors; CD45, receptor-like tyrosine phosphatase, which regulates the action of the Src family kinases. The bottom right table presents the names of the genes and proteins. The following molecules, proteins and organelles are depicted in dark grey: P, phosphorylation; PIP_2_, phosphatidylinositol-4,5-biphosphate; PIP_3_, inositol-1,4,5-trisphosphate; PDK1, 3-phosphoinositide-dependent protein kinase-1; p85, regulatory subunit of PI3K; S6, S6 ribosomal protein; Src, family of kinases, which triggers BCR signalling by phosphorylation of Igα and Igβ; ER, endoplasmic reticulum.

Various lines of evidence indicate that both genetic and epigenetic factors play a role in the development of primary immunodeficiencies^30^. Despite enormous efforts to elucidate the genetic basis of CVID, its molecular basis remains elusive for most patients. Several factors and pathways relevant to B-cell biology have been associated with CVID in genetic studies. Our analysis here has revealed additional elements of these pathways that are altered at the DNA methylation level (Fig. 5). An important target for alterations in CVID is the BCR signalling pathway. BCRs initiate and control several key processes, such as proliferation, differentiation, migration, activation and survival of B cells, and signalling through this receptor is important not only during the first steps of B-cell differentiation in the bone marrow, but also for the proper function of fully differentiated B cells^31^. Although the BCR has not been reported to be genetically altered in CVID patients, and is by itself the source of a separate group of PIDs, there is evidence of alterations in several molecules that are necessary to its function in this disorder. For instance, genes that encode molecules such as CD19, CD21, CD81 and CD20^32^,^33^,^34^,^35^ are mutated in some CVID patients. CD19, CD21 and CD81 make up the B-cell co-receptor complex, whose function is to decrease the threshold of B-cell activation, conferring an enhanced response against pathogens at low concentrations. Specifically, CD81 is necessary for the expression of CD19, while CD21 is a receptor for the complement component C3d. Moreover, CD19 is responsible for BCR signal enhancement mainly through the activation of PI3K signalling.

In our study, one of the most interesting genes displaying increased DNA methylation in memory B cells from CVID individuals was *PIK3CD*. This gene encodes p110δ, the catalytic subunit of one of the PI3K isoforms, a master regulator of B-cell signalling due to its role in the transduction of signals from different molecules and receptors. Through the generation of inositol-1,4,5-trisphosphate 3 (PIP3), p110δ recruits and activates important proteins that drive the downstream effects of BCR signalling^26^. Mutations in this gene have been described for several PIDs^10^,^36^,^37^.

One of the downstream effects of PI3K signalling is the increase in the intracellular Ca^2+^ levels to generate a ‘Ca^2+^ signal'. PIP3 can induce the release of the calcium stores from the endoplasmic reticulum to the cytosol. Another downstream effect of PI3K signalling is the activation of the AKT/mTORC/SP6K axis. PIP3 promotes the activation of AKT, which in turn phosphorylates and activates the mTORC complexes. The activated mTORC complexes then phosphorylate and activate different substrates, such as the S6K2 kinase (encoded by *RPS6KB2*; Fig. 5). We have also found this gene to be hypermethylated in memory B cells in CVID patients. The S6K2 kinase has several substrates; however, its main function is to phosphorylate the ribosomal protein S6, promoting cell growth and protein synthesis.

Once the Ca^2+^ signal has been initiated, other proteins become involved in the enhancement and maintenance of the raised Ca^2+^ levels. Although Coronin 2, encoded by *CORO1B*, also hypermethylated in CVID memory B cells, has not been related to B cells directly, it has been reported that one member of its family, *CORO1A*, has an important role in the immune system through Ca^2+^ mobilization mediated by actin remodelling changes. It is possible that *CORO1B* has similar roles in the B-cell context, but this remains to be determined. Other molecules with an important role in calcium signalling are the potassium channels, like those encoded by other genes hypermethylated in CVID B cells, *KCNN4* and *KCNC4* (Fig. 5). They are responsible for maintaining the elevated calcium levels through the mobilization of K+ ions to the extracellular medium.

B-cell differentiation and activation require high rates of protein synthesis and proliferation. In normal conditions both may provide an apoptotic stimulus, requiring the action of anti-apoptotic molecules such as BCL2-XL (Fig. 5), encoded by the *BCL2L1* gene, and which are also hypermethylated in CVID B cells. This protein maintains the mitochondrial potential membrane and blocks the release of cytochrome *c* from mitochondria.

Finally, at the end of BCR signalling there are the transcription factors, which can transform the signals received at the cell surface into gene expression changes that enable the cell to adapt and respond. To ensure that the proper gene expression pattern is achieved for the different stimuli, it is important not only for several transcription factors to be activated but also for those that are not required to be blocked. E2A (encoded by another hypermethylated gene in CVID memory B cells, namely *TCF3*; Fig. 5) and NF-κB are two examples of transcription factors activated during BCR signalling, while FOXO transcription factors are blocked after BCR activation.

Our findings regarding the impaired ability to demethylate genes during the transition from naive or memory cells reinforces the notion emerging from various studies that CVID individuals are deficient in differentiating towards memory B cells. For instance, we have known for over 40 years that CD73 activity is reduced in B lymphocytes of patients suffering from immunodeficiency syndromes^38^,^39^ and we now know that CD73-dependent adenosine generation favours class-switch recombination, endowing the B cell with an intrinsic control of differentiation towards immunoglobulin class-switched plasma cells^40^. As mentioned above, CVID patients have severely deficient levels of switched memory B cells^41^. These altered proportions of B-cell subsets are also reflected in the cell counts in the cohort of CVID individuals analysed in this study. Interestingly, at the DNA methylation level, genes such as *PIK3CD*, *RPS6KB2*, *KCNN4*, *KCNC4*, *CORO1B* and *BCL2L1* are hypermethylated in memory B cells of CVID individuals relative to healthy individuals, or, in other words, they do not undergo the demethylation in the transition from naive to memory cells observed in healthy individuals. This could also be interpreted as meaning that if the DNA methylation patterns for these genes in memory cells from CVID individuals were reminiscent of those from naive cells, their ability to respond/maintain appropriate responses could therefore be diminished.

Finally, our investigation demonstrates the utility of twin studies for identifying epigenetic changes in complex diseases. MZ twins share their entire genotype, including potentially specific susceptibility gene variants. Therefore, our results reinforce the notion that, for a particular genetic background, DNA methylation changes are related to the onset of CVID. However, we cannot distinguish whether these alterations in the DNA methylation levels are a cause or a consequence; in other words, whether the inability of certain CVID individuals to demethylate these B-cell genes, perhaps influenced by the environment, triggers the onset of CVID; or whether other causes mark the development of CVID (for instance, in one twin in contrast with his healthy sibling) and alterations in DNA methylation are an associated manifestation of CVID, which are associated with deficient B-cell function.

A potential constraint on the use of this approach arises from the fact that somatic mutations, for instance, in the haematopoietic lineage, may arise during development and that even MZ twins may not be not genetically identical in some tissues. In addition, MZ monochorionic twins exchange blood through shared vascular communication. Therefore, MZ twins are a good model system for diseases, such as PIDs, that start to develop after birth, when there is no blood sharing. Our data do not allow us to draw any conclusions about the timing of the DNA methylation changes observed, although it is likely that they are associated with the onset of the disease.

A previous study by our team revealed the existence of DNA methylation differences between MZ twins that are discordant for SLE^23^, reinforcing the notion of an epigenetic component in complex, immune-related diseases. Epigenetic differences in MZ twins become more pronounced with age, supporting the idea that ‘epigenetic drift' plays a role in the divergence of MZ phenotypes^42^. Another epigenetic study on MZ and DZ twins provided further evidence that differences in epigenomes can explain phenotypic differences^43^. The present report, in which the discordance for CVID between a pair of MZ twins is used to identify epigenetic targets, highlights the potential of this strategy for learning about the contribution of epigenetics to immune-related disorders as well as to other complex diseases.

## Methods

### Patients and ethics statement

Human blood samples used in this study were obtained from CVID patients, diagnosed according to established criteria, including one of the siblings of the MZ twin pair and from blood donors. They were collected at the University Hospital Dr Negrín of Gran Canaria (MZ twins discordant for CVID) and the University Hospital La Paz in Madrid (CVID cohort and healthy donors). The blood donors received oral and written information about the possibility that their blood would be used for research purposes, and any questions that arose were then answered. Before giving their first blood sample the donors signed a consent form approved by the Ethics Committee at their corresponding hospital, which adhered to the principles set out in the WMA Declaration of Helsinki. The protocol used to isolate B cells from these donors was approved by IDIBELL's Committee of Biosecurity (CBS) on 5 May 2011 and the Ethics Committee of the University Hospital of Bellvitge (CEIC) on 28 May 2011.

### Sample preparation and isolation of B-cell subsets

Peripheral blood mononuclear cells (PBMCs) from MZ twins were isolated by Lymphoprep (Stem Cell Tech Inc., Vancouver, BC, Canada) density gradient centrifugation. The collected cells were washed twice with ice-cold PBS, followed by centrifugation at 2,000 r.p.m. for 5 min. The CD19+ cells were isolated by positive selection using CD19 MicroBeads (Miltenyi Biotec, Cologne, Bergisch Gladbach, Germany).

For the isolation of CVID and healthy control group B cells, PBMCs were obtained from peripheral blood by Ficoll gradient using Lymphocyte Isolation Solution (Rafer, Zaragoza, Spain). PBMCs were enriched in B cells by negative depletion using CD3 and CD14 Microbeads (Miltenyi Biotec). For the isolation of naive (CD19+CD27−IgD+), unswitched memory (CD19+CD27+IgD+) and switched memory (CD19+CD27+IgD−) B lymphocytes, B-cell-enriched PBMCs were stained with CD19 fluorescein isothiocyanate, CD27 APC (Becton Dickinson, Franklin Lakes, NJ, USA) and IgD PE (Southern Biotech, Birmingham, AL, USA). Cells were sorted on a FACS Aria (Becton Dickinson). Purity check was >95% for all selected fractions. Purified samples were pelleted and stored at −80 °C.

### DNA isolation and bisulfite modification

Pelleted cells were resuspended in 750 μl of lysis buffer (50 mM Tris pH 8.8, 10 mM EDTA pH 8.3, 100 mM NaCl, 1% SDS). In all, 50 μl of proteinase K (10 mg ml^−1^) and 1 μl of glycogen (20 mg ml^−1^) were added, and then incubated overnight at 37 °C. After that time the degraded proteins were precipitated by adding 340 μl of 5 M NaCl and centrifuged at maximum speed for 15 min. The supernatant was collected and 450 μl of 100% isopropanol was added. The isolated DNA was then transferred to a new tube and washed with 75% ethanol. The DNA was resuspended in 10 μl of DNAse-free water. For the subsequent DNA methylation analysis the DNA samples were bisulfite-converted using the EZ DNA methylation kit (Zymo Research, Orange, CA, USA) following the manufacturer's instructions.

### DNA methylation profiling using universal bead arrays

The methylation profiles of bisulfite-modified DNA of CD19+ cells from MZ twins discordant for CVID were compared using the Infinium HumanMethylation450 BeadChips (Illumina Inc., San Diego, CA, USA). This platform allows the interrogation of >485,000 methylation sites per sample at single-nucleotide resolution, and comprises an average of 17 CpG sites per gene in the 99% of RefSeq genes. Ninety-six percent of CpG islands are covered, with additional coverage in CpG island shores and the regions flanking them. The samples were hybridized in the array following the manufacturer's instructions. Each methylation data point is obtained from a combination of the Cy3 and Cy5 fluorescent intensities from the M (methylated) and U (unmethylated) alleles. Background intensity computed from a set of negative controls was subtracted from each data point. For further analysis we used the *β* value of each CpG, which is the ratio of the methylated probe intensity to the overall intensity (sum of methylated and unmethylated probe intensities). The value of *β* ranges from 0 (non-methylation) to 1 (total methylation).

### Selection of differentially methylated CpG candidates

We estimated the *β* values following what was defined as the optimal three-step pipeline in ref. 44. For the differential methylation we transformed *β* values into *M* values (as suggested in ref. 45) and used limma Bioconductor package for the differential methylation analysis. Given that (1) technical variability was similar to biological variability, (2) many DNA methylation changes were expected to be mild and (3) the reduced number of samples (one twin pair), we made a conservative selection of probes as candidate differentially methylated sites: (i) 10% or greater difference in the *β-*value DNA methylation between MZ twins, (ii) *P* value smaller than 0.01 and (iii) in the three replicates the *β*-value estimated of one twin was always larger or always smaller than that of the other twin. The *P* value obtained in (ii) computes the difference of methylation between twins by using the three technical replicates per individual; we only made use of this contrast as a selection criterion that allows the identification of CpG sites where the difference in methylation is larger than the technical variation. This selection criterion returned a selection of 311 differentially methylated candidate sites. Those sites are not statistically significant if FDR or FWER is considered; however, they provide a robust initial candidate selection.

### Gene ontology analysis

The Gene Symbol of probes differentially methylated were uploaded in the AmiGO database^46^. We performed GO enrichment analysis focusing on Biological Process categories on the basis of genes that displayed at least twofold differential methylation compared with each probe. To filter non-enrichment GO terms we applied a threshold *P* value and adjusted *P* value of less than 0.05. The hypermethylated and hypomethylated genes were also analysed with Ingenuity programme and GO database. The hypermethylated genes were selected on the basis of their constant presence in relevant B-cell signalling pathways, their implication in common regulatory networks and functions related to BCR signalling.

### Gene expression data analysis

We compared the expression array values from GSE17269 Affymetrix Human Genome U133 Plus 2.0 Array microarray^29^. Specifically, we compared expression data of B cells from CVID donors and healthy donors. To obtain differential expression values between diseases and control samples, we used Bioconductor package Affy functions to read Affymetrix samples with CEL format. The Limma package was then used to estimate the differential expression of each probe ID, using the same parameters as estimated in the original study. Raw differential expression data were selected on the basis of them showing a lower than 0.5-fold or greater than 1.5-fold change and a *P* value less than 0.05. Finally, the overlap of differentially methylated and differentially expressed gene values was evaluated using Gene Symbol as ID and the *n*-fold change as numeric values.

To test the expression of selected genes, we isolated RNA from naive, unswitched and switched memory cells using the RNeasy Plus Micro Kit (Qiagen, Venlo, Limburg, the Netherlands) adding MS2 RNA (Roche, Basel, Switzerland) to stabilize the RNA template. We reverse-transcribed total RNA using Transcriptor First Strand cDNA Synthesis Kit from Roche Diagnostics. QRT–PCR analysis was performed in a PCR Real-Time LightCycler 480 (Roche) with SYBR green. Primer sequences are listed in Supplementary Table 2.

### Bisulfite pyrosequencing

To validate the results from the DNA methylation array, we selected seven differentially methylated CVID-relevant genes with which to perform the pyrosequencing analysis. We analysed the samples of CD19+ cells from MZ twins and a panel of 84 samples corresponding to three different cell populations: naïve, unswitched and switched B cells, of a panel of CVID patients and healthy donors. Biotinylated amplicons for each gene were generated with PCR using the HotStart Taq DNA polymerase PCR kit (Qiagen). Specific primers were designed using the PyroMark Assay Design Software (QIAGEN version 2.0.01.15). Pyrosequencing reactions were performed and DNA methylation quantified with the Pyromark Q24 system (Qiagen). Results from bisulfite pyrosequencing are presented as the percentage of methylation. Raw bisulfite sequence data are provided as Supplementary Data 2. Primer sequences are listed in Supplementary Table 2.

### Chromatin immunoprecipitation assays

Chromatin immunoprecipitation (ChIP) assays were performed using LowCell# ChIP kits (Diagenode, Seraing, Belgium) following the manufacturer's instructions on naive, unswitched and switched memory cells crosslinked with 1% formaldehyde for 10 min. We used the following antibodies: H3K4me3 (no. CS200580, Millipore, Billerica, MA, USA) and H3K27me3 (ref: 07–449, Millipore). Primer sequences are listed in Supplementary Table 2.

### Statistical analysis

The B-cell subsets from CVID patients and healthy donors were compared using a Student's *t*-test and ANOVA. *P* values less than 0.05 were considered statistically significant.

## Additional information

**Accession codes:** Methylation array data for this publication have been deposited in NCBI's Gene Expression Omnibus and is accessible through GEO Series accession number GSE63849.

**How to cite this article:** Rodríguez-Cortez, V. C. *et al.* Monozygotic twins discordant for common variable immunodeficiency reveal impaired DNA demethylation during naive-to-memory B-cell transition. *Nat. Commun.* 6:7335 doi: 10.1038/ncomms8335 (2015).

## Supplementary Material

Supplementary Tables Supplementary Tables 1-2

Supplementary Data 1 CpG sites differentially methylated between the CVID and healthy sibling of a monozygotic twin pair. Columns include β values for the three independent replicates (R1, R2, R3) of the CVID sibling (D, E and F) and healthy/control sibling (G, H, I), as well as the average β values (J, K). The difference b value between CVID and Healthy/control is also presented (L). The rest of columns include: CpG id code (M), chromosome (N), genome coordinates (O), UCSC_RefGene_Name (P), position of the CpG within the gene (Q), location pf the CpG island, where applicable (R), and relation to UCSC CpG Island (S). Hypermethylated genes are in sheet 1 and hypomethylated genes in sheet 2.

Supplementary Data 2 Number of cells in each population and donor and DNA methylation percentages. Number of cells isolated for each cell subpopulation (naïve, unswitched and switched memory B cells) are presented for all control and CVID individuals studied. The percentage of methylation for the selected CpG site of each gene is also shown, as obtained from bisulfite pyrosequencing.

## Figures and Tables

**Table 1:** Selected hypermethylated genes in B cells when comparing the CVID twin versus his healthy sibling.

**Gene**	**CpG number**	**Localization**	**ΔBeta value**	**Relevance of gene products in B-cell context**
*BCL2L1*	cg13989999	Body	0.13	The longest isoform encoded by this gene, BCL2-X_L_, is an apoptotic inhibitor and plays a critical role in regulating cell survival in the GC. It has been reported that T lymphocytes from CVID patients are more sensitive to apoptosis due to deregulation of this molecule.
*TCF3*	cg26615224	Body	0.13	Important transcription factor that mainly acts as gene activator. It is required for normal T- and B-cell development.
*PIK3CD*	cg03265564	5′ UTR	0.11	The p110δ catalytic subunit is mainly expressed in haematopoietic cells. This molecule mediates the chemokine-induced migration, BCR signalling, BCR-induced proliferation and differentiation into Antibody-producing cells
*RPS6KB2*	cg03559915	Body	0.15	This kinase is a downstream signalling protein of the mTOR pathway. S6K2 regulates cell growth and protein synthesis
*KCNN4*	cg26890181	TSS1500	0.11	It is upregulated during B-cell activation and helps to maintain the enhanced cytosolic Ca^+2^ levels during signal transduction
*KCNC4*	cg26189021	Body	0.14	This K+ channel modulates the membrane potential directly by regulating the generation of cytosolic Ca2+ signals. KCNC4 channels are key regulators in the stress response of irradiated leukaemia cells
*CORO1B/PTPRCAP*	cg12044599	Body*/*TSS1500	0.17	Coronin 2, the protein product of the *CORO1B* gene, has been implicated in actin-based processes such as cell migration. Coronin1A, another member of coronin protein familiy, has been related to T-cell survival and has been associated with T-cell immunodeficiencies
	cg23468927	Body*/*TSS1500	0.15	The protein encoded by *PTPRCAP* is required for normal antigen-receptor signalling and function in lymphocytes. Its association with the protein phosphatase CD45 seems to modulate signal transduction by regulating the CD45–LCK interaction
*CCL5*	cg10315334	5′ UTR	0.13	
*XCL1*	cg21872093	TSS1500	0.13	This chemokine is important in the regulation of T and B lymphocytes and neutrophil trafficking
*WNT5A*	cg24049183	Body	0.17	WNT5A protects isolated GC B cells from apoptosis by initiating the noncanonical b-catenin-independent signalling pathway
*AKT3*	cg24455383	Body	0.14	AKT, as the major effector downstream of PI3K signalling pathway, plays a key role in peripheral B-cell maturation and survival
*RPTOR*	cg00701918	Body	0.15	This component of the mTORC1 complex is one of the main targets of AKT. It is required for proliferation of splenic B cells and promotes B-cell responses to LPS in the absence of PI3K activation
*IKBKE*	cg26859016	TSS1500	0.11	IKBKE is a noncanonical IKK family member that plays an important role in the regulation of inflammatory signalling pathway. This kinase activates NF-κB and is able to activate AKT in a PI3K-independent way.
*STK11*	cg08317252	Body	0.11	It has been reported that this master kinase is important for cessation of the GC reaction, plasma cell differentiation and suppression of tumorigenesis
*DUSP2*	cg02431562	Body	0.13	This phosphatase is involved in the regulation of a number of MAP kinase-dependent physiological processes occurring during the proliferation and differentiation of haematopoietic cells
*TNFRSF10A*	cg23303108	TSS1500	0.11	Receptor of the pro-apoptotic protein TRAIL, which has been involved in apoptosis, necessary to eliminate primary plasma cells after the synthesis and secretion of large amounts of antibodies
*HDAC4*	cg11231069	Body	0.12	Chromatin modifier that is recruited by the transcriptional repressor BCL-6 to regulate lymphocyte function, survival and differentiation
*MTA3*	cg06342490	TSS1500	0.15	MTA3 is a subunit of the transcriptional co-repressor Mi-2/NuRD, with a prominent role in B-cell fate determination through its interaction with BCL-6

CVID, common variable immunodeficiency; GC, germinal centre; LPS, lipopolysaccharide; PI3K, phosphatidylinositol 3-kinase; TSS, transcription start site; UTR, untranslated region.

**Table 2:** DNA methylation levels in selected genes for CVID and healthy cohorts in naive, unswitched and switched memory B cells.

**Gene**	**Naïve**		**Unswitched**		**Switched**	
	**AVG %Meth**	**s.d.**	**AVG %Meth**	**s.d.**	**AVG %Meth**	**s.d.**
*BCL2L1*						
Control	79	22	49	25	13	6
CVID	80	31	62	25	21	24
						
*TCF3*						
Control	87	7	42	20	21	10
CVID	77	29	62	32	32	25
						
*PIK3CD*						
Control	80	23	33	18	18	16
CVID	82	23	54	29	31	26
						
*RPS6KB2*						
Control	87	13	22	28	14	22
CVID	76	33	66	40	35	45
						
*KCNN4*						
Control	93	8	35	28	8	9
CVID	86	15	53	37	11	9
						
*KCNC4*						
Control	87	7	40	20	11	8
CVID	84	17	41	41	59	37
						
*CORO1B/PTPRCAP*						
Control	81	18	46	38	26	25
CVID	87	11	72	31	64	39
Control	81	31	21	29	18	23
CVID	88	15	44	40	31	38

Avg, average; CVID, common variable immunodeficiency; Meth, DNA methylation.

DNA methylation levels are measured by bisulfite pyrosequencing and represented as percentage.
