# Supplementary material for: Monozygotic twins discordant for common variable immunodeficiency reveal impaired DNA demethylation during naïve-to-memory B-cell transition
Source: Nat Commun. 2015 Jun 17;6:7335. doi: 10.1038/ncomms8335 (PMC4557293; doi:10.1038/ncomms8335)
Supplement: Supplementary Tables — 1-2 [file ncomms8335-s1.pdf]

Supplementary Table 1. Analysis of variance (ANOVA)

		SS	DF	MS	F	S(p)
BCL2L1, naïve	Between	6.857	1	6.857	0.009	0.925
	Within	19,739.000	26	759.192		
	Total	19,745.857	27			
BCL2L1, unswitched	Between:	1,158.857	1	1,158.857	1.854	0.185
	Within:	16,250.000	26	625.000		
	Total:	17,408.857	27			
BCL2L1, switched	Between:	438.857	1	438.857	1.263	0.271
	Within:	9,036.000	26	347.538		
	Total:	9,474.857	27			
TCF3, naïve	Between:	685.714	1	685.714	1.355	0.255
	Within:	13,154.000	26	505.923		
	Total:	13,839.714	27			
TCF3, unswitched	Between:	2,742.857	1	2,742.857	3.609	**0.069**
	Within:	19,760.000	26	760.000		
	Total:	22,502.857	27			
TCF3, switched	Between:	829.714	1	829.714	2.059	0.163
	Within:	10,475.000	26	402.885		
	Total:	11,304.714	27			
PIK3CD, naïve	Between:	27.429	1	27.429	0.052	0.822
	Within:	13,754.000	26	529.000		
	Total:	13,781.429	27			
PIK3CD, unswitched	Between:	3,024.000	1	3,024.000	4.860	**0.037**
	Within:	16,179.000	26	622.269		
	Total:	19,203.000	27			
PIK3CD, switched	Between:	1,158.857	1	1,158.857	2.326	0.139
	Within:	12,956.000	26	498.308		
	Total:	14,114.857	27			
RPS6KB2, naïve	Between:	829.714	1	829.714	1.186	0.286
	Within:	18,194.000	26	699.769		
	Total:	19,023.714	27			
RPS6KB2, unswitched	Between:	13,275.429	1	13,275.429	10.580	**0.003**
	Within:	32,624.000	26	1,254.769		
	Total:	45,899.429	27			
RPS6KB2, switched	Between:	3,024.000	1	3,024.000	2.202	0.150
	Within:	35,699.000	26	1,373.038		
	Total:	38,723.000	27			
KCNN4, naïve	Between:	336.000	1	336.000	2.142	0.155
	Within:	4,079.000	26	156.885		
	Total:	4,415.000	27			
KCNN4, unswitched	Between:	2,221.714	1	2,221.714	1.981	0.171
	Within:	29,159.000	26	1,121.500		
	Total:	31,380.714	27			
KCNN4, switched	Between:	61.714	1	61.714	0.762	0.391
	Within:	2,106.000	26	81.000		
	Total:	2,167.714	27			
KCNC4, naïve	Between:	61.714	1	61.714	0.329	0.571
	Within:	4,874.000	26	187.462		
	Total:	4,935.714	27			
KCNC4, unswitched	Between:	6.857	1	6.857	0.006	0.939

	Within:	29,615.000	26	1,139.038		
	Total:	29,621.857	27			
KCNC4, switched	Between:	15,798.857	1	15,798.857	19.340	**0.000**
	Within:	21,239.000	26	816.885		
	Total:	37,037.857	27			
CORO1B, naïve	Between:	246.857	1	246.857	1.193	0.285
	Within:	5,379.000	26	206.885		
	Total:	5,625.857	27			
CORO1B, unswitched	Between:	4,635.429	1	4,635.429	3.978	**0.057**
	Within:	30,299.000	26	1,165.346		
	Total:	34,934.429	27			
CORO1B, switched	Between:	9,901.714	1	9,901.714	8.671	**0.007**
	Within:	29,690.000	26	1,141.923		
	Total:	39,591.714	27			
CORO1B, naïve	Between:	336.000	1	336.000	0.626	0.436
	Within:	13,946.000	26	536.385		
	Total:	14,282.000	27			
CORO1B, unswitched	Between:	3,627.429	1	3,627.429	2.836	**0.104**
	Within:	33,251.000	26	1,278.885		
	Total:	36,878.429	27			
CORO1B, switched	Between:	1,158.857	1	1,158.857	1.096	0.305
	Within:	27,479.000	26	1,056.885		
	Total:	28,637.857	27			

SS, sum of squares; Df, degrees of freedom; MS, mean of squares; F, Fisher-Snedecor test; S, significance: when p<0.1 statistically significant difference between control and CVID groups.

Supplementary Table 2. Primers for bisulfite pyrosequencing, mRNA analysis and ChIP analysis

	Amplification primers			
**Gene**	**Forward Primer**	**Reverse Primer**	**Pyrosequencing Primer**	**Analyzed CpG**
BCL2L1	[Btn]AGTAGTAAAGTAAGYGTTGAGGGAGGTAGG	CACAATACRACCCCAATTTACCCCATCC	AATATCAAATCACTAAATACCC	cg13989999
CORO1B / PTPRCAP	[Btn]AGTTTTGGAGGTTGAGGAGTG	CCCAACACCACCCCTACCTA	AAATCCCRCTACTTACTAAACAC	cg12044599
	[Btn]AGTTTTGGAGGTTGAGGAGTG	ACCAAAACCACACCTAAAACTC	CATAACRAACCRACTATCAAACAAC	cg23468927
KCNC4	GTTYGTAGTATTGTGGGGTGGTG	[Btn]ATCAACTCAATTCACTTTCCATTTATA	GTAGTGYGTTATGATAGGAGTAAAA	cg26189021
KCNN4	ATGTTGTTTTGTGTGGTTAGAATAT	[Btn]AAAAACATACCTATAATAACCCCCAATC	ATTATTATTGCRAGTATTTGTG	cg26890181
PIK3CD	[Btn]ATAAGGATTGTTTYGGTGTTGTTATTGTA	AACTCAAATCCAACCTACTAACTATTC	ACTAATACATCTATCCATTACA	cg03265564
RPS6KB2	GGGGTTTGAGGTTTGTGGGATTA	[Btn]AACCCTCCTTAATACTATCCAAAACA	AGGTTTGTGGGATTAG	cg03559915
TCF3	[Btn]TATAGGTTTTYGAGGGATTATAGTTGGT	ATCAAAAACCRACCTCTCAAATCACTT	CCACCCCCCATACCC	cg26615224

[Btn] : Biotinylated Primer

Primers for mRNA analysis	**Forward Primer**	**Reverse Primer**
BCL2L1	GCGGCTGGGATACTTTTGT	AGCGGTTGAAGCGTTCCT
TCF3	GCTCAGTGACCTCCTGGACT	CAAGACCTGAACCTCCGAAC
PIK3CD	ACCTCAGCACCATCAAGCA	GGTTGATGCAGGTGAACACA
KCNN4	GGAACTGGCATTGGACTCAT	TGCTGATCGTGCATTTAACC
PTPRCAP	ACTTCTCGCTCGACACAGC	CATCCCGAGCCCTAAGGT
CORO1B	CCCGGTTCTACAAACTGCAT	CTGTGTCGGGGTACAGATCA

Primers for ChIP analysis	**Forward Primer**	**Reverse Primer**
BCL2L1	CAGTAAAGCAAGCGCTGAGG	GTGATGTGGAGCTGGGATGT
PI3KCD	acaaggattgtctcggtgttg	ataaatgagtgtacctagcactttcac
TCF3	AGCACTGGCCTCGgtgag	aggaggaccaggagagatgg
